# Longitudinal Slit Procedure in Addition to Negative Pressure Wound Therapy for a Refractory Wound With Exposed Achilles Tendon

**Published:** 2015-03-18

**Authors:** Erika Ohata, Shunsuke Yuzuriha, Yoshito Mishima, Kiyoshi Matsuo

**Affiliations:** ^a^Department of Plastic and Reconstructive Surgery, Shinshu University School of Medicine, Matsumoto, Japan; ^b^Department of Plastic and Reconstructive Surgery, Nagano Red Cross Hospital, Nagano, Japan

**Keywords:** negative pressure wound therapy, Achilles tendon, longitudinal slit, burn, foot

## Abstract

**Objective:** This case report reviews features of negative pressure wound therapy, particularly for the exposed Achilles tendon, and describes an additional effective procedure. **Methods:** An 87-year-old man presented with a soft-tissue defect measuring 3×5 cm with the exposed Achilles tendon as a sequela of deep burn. The condition of his affected leg was ischemic because of arteriosclerosis. We used negative pressure wound therapy and made 2 longitudinal slits penetrating the tendon to induce blood flow from the ventral side to the dorsal surface. **Results:** By this combination therapy, the surface of the exposed Achilles tendon was completely epithelialized and the tendon was spared without disuse syndrome. **Conclusions:** The authors conclude that this combination therapy is useful for covering the widely exposed tendon in aged patients.

Deep burns of the foot occasionally cause exposure of tendons and create a challenge for wound coverage because skin grafts cannot take without an intact paratenon.^1^ We have successfully used negative pressure wound therapy (NPWT)^2^ in combination with the additional surgical option in a case of widely exposed Achilles tendon in an elderly patient with burn injury.

This case report reviews features of NPWT, particularly for the exposed Achilles tendon, and describes the additional longitudinal slits procedure penetrating the tendon required to close the impaired wound with the exposed tendon.

## METHODS/CASE REPORT

An 87-year-old man sustained 5% total body surface area full-thickness flame burn to his left lower leg and foot. Initial surgical debridement and autologous 1:3 meshed split-thickness skin grafts were performed to his left lower extremity on day 7 after the injury. Seven days after the operation, he presented with a soft-tissue defect measuring 3×5 cm with the exposed Achilles tendon. The condition of the affected leg was somewhat ischemic because of arteriosclerosis, which was judged to be due to calcification of blood vessels as confirmed by computed tomography. On day 23 after the first operation, NPWT (the VAC device; KCI, San Antonio, TX) was applied to the wound covered with a nonadherent wound contact dressing composed of a 100% polyester crosswise open weave (Urgotul; Nitto, Tokyo, Japan) after superficial debridement of the Achilles tendon. We used the black polyurethane foam and chose a continuous negative pressure of 125 mm Hg. The dressing was changed twice a week.

**Figure 1:**
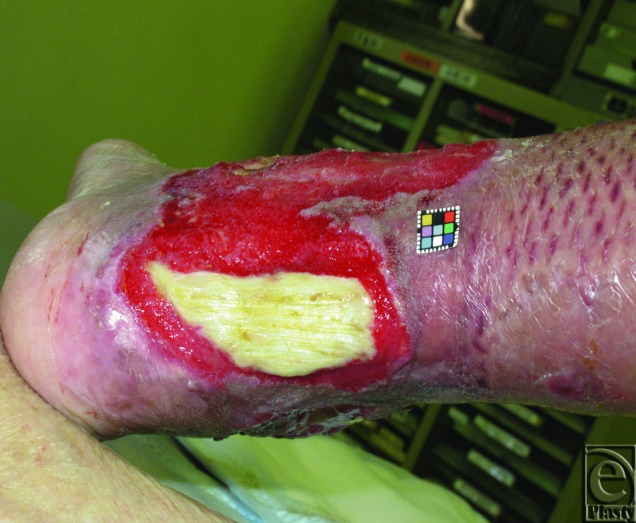
Appearances on day 40 after injury. After 10 days of continuous negative pressure wound therapy.

**Figure 2:**
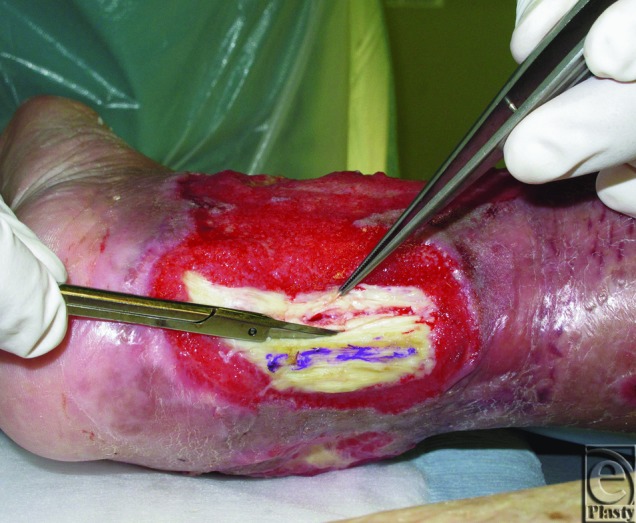
Longitudinal slit procedure.

After 10 days of continuous NPWT, no granulation tissue was observed on the Achilles tendon (Fig 1). We made 2 longitudinal slits penetrating the tendon to induce blood flow from the ventral side to the dorsal surface deliberately (Fig 2). Some shredded foam was placed into the slits to keep them open, and NPWT was continued. After 28 days of continuous NPWT, the Achilles tendon was completely covered with well-vascularized granulation tissue and the patient was discharged. He received standing and walking rehabilitation throughout the wound treatment. He left our hospital on foot as an ambulatory patient.

**Figure 3:**
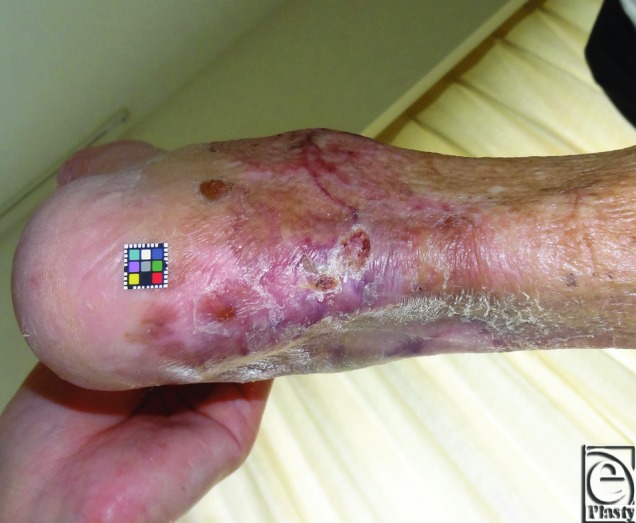
Five months after leaving the hospital.

Epithelialization was completed by ordinary topical therapy with ointment at home a few months after leaving hospital (Fig 3).

## DISCUSSION

Treatment of wound closure of exposed tendons is challenging. Traditionally, flap surgical procedures are used, for example, local flaps, cross-leg flaps, and free flaps.^3^^-^9 Local flaps are not always available for burned ankles with an exposed Achilles tendon because the presence of surrounding burn usually eliminates any prospect of local tissue transfer.^9^ Cross-leg flaps are available if the contralateral leg has no wound, although patients must be prevented from moving their legs for several weeks. Free flaps have proven to be a reliable reconstructive option in burn care and are available for tendon coverage if intact recipient vessels can be confirmed.

In the present case, there was no intact surrounding tissue available for the local flap and the calcification of blood vessels was confirmed. Furthermore, long rest of the legs may cause disuse syndrome, especially in elderly patients. Therefore, we chose NPWT as an option to achieve early wound closure without forcing long-term rest of the legs.

**Figure 4:**
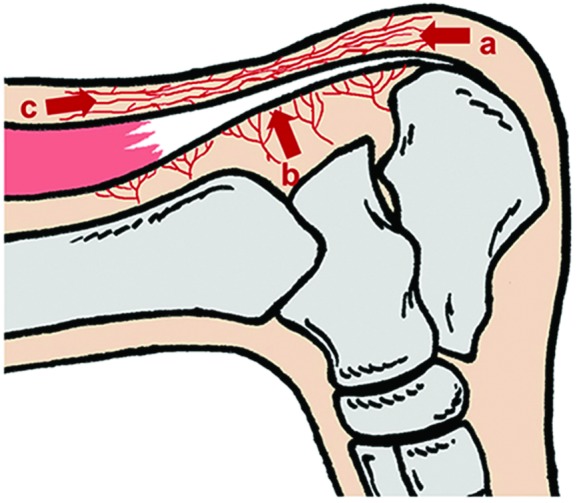
Blood flow feeding the paratenon: (*a*) from the osteotendinous junction; (*b*) from the mesotenon; and (*c*) from the musculotendinous junction.

In some successful case reports of NPWT dealing with Achilles tendon exposure, vascularized tendons were observed after a few weeks of NPWT.^1^ However, we did not observe any granulation tissue formation on the Achilles tendon even after 10 days of NPWT. This protracted wound healing was thought to be mainly due to the completely damaged paratenon at the surface of the Achilles tendon. The healing of tendons requires an intact paratenon to provide nutrition to the tendon tissue. The blood flow into the paratenon is supplied from the surrounding tissue (Fig 4). Furthermore, the direct blood supply to the tendon comes from a vascular cascade that enters on its ventral surface.^10^

In the present case, as the paratenon at the surface was completely absent, the formation of granulation tissue should be promoted by blood supply only from the ventral surface and sides of the tendon. Continuous NPWT alone was insufficient to stimulate granulation from the sides. Therefore, we made longitudinal slits penetrating the tendon to induce blood flow to the dorsal surface from the ventral surface. This procedure is based on the same theory as the trephination of bone to cover exposed bone surface.^11^^,^^12^ By combining this surgical procedure and NPWT, we were able to form a granuloma at the surface of the Achilles tendon and spare the tendon itself.

When we try to apply NPWT to wound with the exposed tendon, we have to consider optimum condition regarding wound contact materials such as Integra (Life Sciences Inc., Plainsboro, NJ),^13^^,^^14^ open-cell foam,^15^ pressure level,^15^ and pressure pattern^16^ in an attempt to fit the therapy to the patient.
